# Measuring change in school-based practices that promote children’s healthy eating and active living: a psychometric study

**DOI:** 10.1186/s12966-026-01921-0

**Published:** 2026-04-17

**Authors:** Carolyn D. Rider, Ramsha Baig, Janice Kao, Sridharshi C. Hewawitharana, Gail Woodward-Lopez, Miranda Westfall Brown

**Affiliations:** Nutrition Policy Institute, Division of Agriculture and Natural Resources, University of California, 1111 Franklin Street, Oakland, CA 94607 USA

**Keywords:** School health promotion, Child health, Nutrition policies, Healthy eating, Physical activity, Surveys and questionnaires, Sensitivity, Psychometrics

## Abstract

**Background:**

Public health interventions targeting youth nutrition and physical activity often focus on changing policies, systems, and environments (PSE) in schools. To support rigorous evaluation of PSE efforts, this study examined whether the School Site-Level Assessment Questionnaire (SLAQ), a self-assessment instrument for schools serving grades kindergarten through 12 (K-12), was sufficiently sensitive to measure changes in schools’ nutrition and physical activity practices in response to interventions.

**Methods:**

This longitudinal, observational study included 69 K-12 schools in low-income California communities that completed School SLAQs in two consecutive school years (2022 and 2023) and reported PSE and/or nutrition education interventions in school year 2022. Wilcoxon signed-rank and paired t-tests compared changes in nutrition and physical activity practices measured by the School SLAQ between years among schools with relevant interventions.

**Results:**

Statistically significant increases in median nutrition (by 0.06, *p* < 0.001; effect size [ES] = 0.42) and PA (by 0.07, *p* < 0.001; ES = 0.64) overall domain scores were observed among schools implementing corresponding interventions. Statistically significant increases were also observed in six of nine practice areas: school meal and beverage quality (by 0.04 [95% CI: 0.002–0.07]), meal environment and promotion (by 0.04 [95% CI: 0.01–0.08]), non-meal food and beverage quality and promotion (by 0.09 [95% CI: 0.05–0.13]), non-physical education physical activity opportunities (by 0.09 [95% CI: 0.02–0.17]), physical activity facilities (by 0.07 [95% CI: 0.03–0.12]), and nutrition education (by 0.10 [95% CI: 0.01–0.18]), among schools implementing corresponding interventions.

**Conclusion:**

The School SLAQ is, to our knowledge, the only tool demonstrated to measure changes in the K-12 school nutrition and physical activity environment. This comprehensive and valid instrument can be a valuable tool for schools and public health partners when planning and evaluating the effectiveness of school-based PSE interventions, as well as measuring the impact of policy initiatives.

**Supplementary Information:**

The online version contains supplementary material available at 10.1186/s12966-026-01921-0.

## Background

Children’s short and long-term health outcomes, including cardiovascular fitness, obesity, and obesity trajectory are impacted by the school environment [1–4]. Accordingly, schools are a common setting for policy, systems, and environmental (PSE) change interventions related to nutrition and physical activity (PA), such as improving the quality of school meals and adding or enriching opportunities for students to be active before, during, or after the school day. PSE interventions related to school meals and non-meal foods and beverages at school have been shown to improve the availability, selection, and intake of healthy foods and reduce consumption of unhealthy foods [5, 6]. Likewise, school-based PSE interventions targeting PA are shown to be effective in improving students’ PA, including moderate to vigorous physical activity, as well as reducing sedentary behaviors [7]. 

Effectively targeted school-based PSE interventions begin with a needs assessment conducted in partnership with school administrators. Buy-in from school leadership during assessment and planning is critical to successful implementation and supportive for long-term sustainability of health promotion programming in schools [8, 9]. Self-assessment tools like the School Site-Level Assessment Questionnaire (SLAQ) or School Health Index can be feasible and effective for engaging school personnel directly in needs assessments and intervention planning [8, 10–12]. However, the School SLAQ is, to our knowledge, the only comprehensive, valid, and reliable self-assessment instrument available to measure school nutrition and PA practices [11]. While other instruments are available to assess school nutrition and PA environments, these tools focus on a limited range of practice areas (e.g., school cafeterias, PA practices), on wellness policy presence rather than implementation, or they have not been validated [12–19]. Questionnaires like the School SLAQ are also commonly used for ongoing assessment and re-evaluation. This is especially important for PSE interventions, because policy changes require ongoing implementation to be effective. However, it is unknown whether any of these instruments, including the School SLAQ, are sufficiently sensitive to measure changes in the school environment over time, such as when reassessing to determine whether a PSE intervention has impacted a policy or practice at a school.

Despite the importance of measuring change over time for rigorous evaluation of PSE interventions, it is uncommon for nutrition and PA policy and practice assessments to undergo sensitivity testing. The current study aimed to address this gap by testing the ability of the School SLAQ to detect change in school nutrition and PA practices in response to PSE interventions at a sample of California schools.

## Materials and methods

### Study sample

California schools were eligible for study participation if they served students in grades K-12 during school years 2021-22 (Year 1) and 2022-23 (Year 2); were eligible to participate in Supplemental Nutrition Assistance Program-Education (SNAP-Ed) based on low-income status, most often defined as at least 50% of students being eligible for free or reduced-price meals; partnered with their local health department to plan and implement nutrition and/or PA PSE interventions; and completed the School SLAQ and other program reporting annually as part of these interventions. Schools were excluded from the study if they did not have available PSE intervention data for Year 1. A minimum of 34 schools was determined to be needed to detect a Cohen’s dz effect size of 0.5, with 80% power and an alpha level of 0.05, via G*Power (version 3.1.9.7). As this study was part of an ongoing health promotion program, participation did not involve compensation beyond any agreement already in place between a school and their local health department partner.

### Materials and procedures

The School SLAQ is a valid, reliable, and comprehensive assessment of nutrition and physical activity practices [11]. Questions vary slightly between elementary and secondary schools to accommodate differences in best practices in the areas of beverages, competitive foods, physical education (PE), recess, and interscholastic sports, with 74 questions scored and analyzed for elementary schools and 76 for secondary schools. Questions are framed as best practices using a standardized statement-and-response format. To optimize item sensitivity, response options indicate degree of implementation on a 5-point Likert-type scale whenever possible; some use a trichotomous scale or yes/no responses. Most items are scored on a 0–4 scale (5-point: 0,1,2,3,4; trichotomous: 0,2,4; yes/no: 0,4); a few items were judged by content experts to merit less weight and are scaled 0–1 or 0–2 accordingly [11]. Item and scale scores represent the extent to which schools implement best practices, with higher scores representing greater adherence. For the full questionnaire, see Supplementary Material 1, Additional File 1.

The School SLAQ was self-administered by one or more school personnel or partners most familiar with their school’s nutrition and physical activity practices; the specific role of individual respondents varied across schools and often included school administrators, cafeteria/food service managers or staff, and teachers. Respondents were instructed to complete the assessment prior to beginning any PSE interventions for that school year, and to only report current practices, not those that may have been planned but not yet implemented. Data were submitted to the research team online using the Survey123 platform. To coordinate responses among school personnel when more than one respondent was required, schools could complete the questionnaire on paper or using a shared, fillable Word or PDF document before submitting final responses in Survey123.

School SLAQ items were categorized into two main domains (nutrition and PA) then further grouped into nine nutrition and PA-related practice areas: wellness policies, school meal and beverage quality, meal environment and promotion, non-meal food and beverage quality and promotion, edible gardens, nutrition education, PE, non-PE PA opportunities, and PA facilities (Table 1). For each year, domain and practice area scores were calculated by summing the score of each item within each category and dividing by the total possible score to get a scaled score from 0 to 1. Schools with missing data for any item within a practice area were excluded from the analysis of that practice area’s score (*n* = 4, 2, 10, 11, 1, 5, 1, 3, and 1 schools for wellness policies, school meal & beverage quality, meal environment and promotion, non-meal food and beverage quality and promotion, garden, nutrition education, physical education, non-PE PA opportunities, and PA facilities practice areas, respectively).

**Table 1:** Policies and practices measured on school SLAQ and corresponding interventions by domain and practice area

Practice area (# schools reporting intervention)	Policies and practices measured on School SLAQ^1^	Corresponding interventions reported in PEARS^2^
**Nutrition domain**		
School meal & beverage quality (56)	• Programs that promote local or regional food for meals • All meal foods and beverages meet National School Lunch Act requirements • Students receive at least one fruit or vegetable with each meal • More than one fruit choice (not including juice) offered at each lunch • More than one vegetable choice offered at each lunch • The only beverages at breakfast are milk and water • The only beverages at lunch are milk and water • Milk served with meals limited to non-flavored and 1% fat or less	• Use of fresh or local produce • Menus/recipes to improve the availability, variety, quality, etc. of healthy food • Availability of healthy beverage options • Food/beverage or nutrition related elements of a school wellness policy • Policy limiting unhealthy foods • Policy increasing healthy foods and beverages
Meal environment & promotion (57)	• Sliced or cut fruit offered with meals • Prominent and attractive displays of fruits and vegetables on service lines • Pre-packaged salads or a salad bar available at lunch • White milk placed for easier access than flavored milk • Free, palatable drinking water available during meal times • Sufficient time for lunch (including sufficient “seat time”) • Lunch between 11:00 am and 1:00 pm • Classroom nutrition education reinforced in the cafeteria • Students help promote the meal program • Students take part in taste tests, surveys, or other methods to determine preferences for menu items • Adequate space for students to sit and eat • Dining facilities are pleasant for students to eat • Drinking water available at no charge to students • Nutritional information is made available to families *Secondary schools only* • Reimbursable grab-and-go lunch option	• Display of meal food/beverages to encourage healthy and discourage unhealthy selections • Improvements to dining/serving facilities to promote healthy selection • Limits on marketing/promotion of less healthy options • Flavor station with healthy seasonings or low-fat dip • Free water access, taste, quality, smell, or temperature • Healthy behavior prompts (e.g., signs or demonstrations) at point of decision • Meal service staff prompts healthy choices • Use of digital platforms to improve access to healthy food • Nutrient labeling on menus, vending machines, etc. • Food/beverage or nutrition related elements of a school wellness policy • Policy increasing healthy foods and beverages
Non-meal food & beverage quality and promotion (56)	• Limits on food and beverage advertising on campus • Limits on unhealthy foods in fundraising efforts • Healthy foods served at school events and celebrations • Pricing incentivizes selection of healthier competitive foods/drinks • Foods and beverages sold during the school day meet competitive food standards • Foods and beverages sold after school meet competitive food standards • Recommended, allowed, and non-allowed beverages sold to students during the school day • Sugar-sweetened beverages sold at school events • Teachers discouraged from serving sugar-sweetened beverages at classroom celebrations • Students allowed to carry refillable water bottles • Guidelines for food brought in for holidays/celebrations provided to parents *Secondary schools only* • Number of venues selling non-meal foods and beverages	• Increase use of healthy food or decrease use of unhealthy food in fundraisers/events • Use of healthy food (or no food) as rewards or during celebrations • Healthier competitive food options • Healthy beverage options • Reduction in amount of competitive foods/beverages • Healthier snack options • Food/beverage or nutrition related elements of a school wellness policy • Policy limiting unhealthy foods • Policy increasing healthy foods and beverages
Garden (25)	• School has access to an onsite or community edible garden • Months the garden was actively growing fruits and/or vegetables • Garden incorporated into nutrition education • How many students tend to the garden • How often students tend to the garden • Produce from the garden is distributed to families • Produce from the garden is used in meals or snacks	• Edible gardens at school • Use of the garden for nutrition education • Opportunities for parents or students to work in the garden • Mechanism for distributing produce from gardens to families or communities • Onsite garden produce is used for meals or snacks
Nutrition education (48)	• Nutrition education offered to students • Proportion of students receiving nutrition education by grade level • Nutrition education follows specific best practices (e.g., skills-based and participatory) • Annual professional development for teachers of nutrition education • Nutrition education offered to students’ parents/caregivers	• Nutrition-related direct education • Professional development opportunities on nutrition
**Physical activity (PA) domain**		
Physical education (PE) (14)	• Minutes of PE students participate in • Sufficient class time spent in moderate to vigorous PA • PE curriculum implemented in alignment with state standards and grade-level benchmarks • Student-teacher ratio in PE comparable to core classes • PE taught by certified and endorsed PE teacher • Annual professional development on PE or PA for teachers of PE	• Increased quantity (minutes) of PE • Quality of PE • Professional development opportunities on PA • PA related elements of a school wellness policy
Non-PE physical activity opportunities (26)	• Teachers encouraged to provide movement breaks throughout the day • Teachers discouraged from using/withholding PA as punishment • Organized PA opportunities offered before and/or after school • Intramural sports or PA clubs offered regardless of gender and ability • PA opportunities offered to parents • Families provided information about enrolling students in before/after-school PA opportunities *Elementary schools only* • Recess provided for all students • Staff actively facilitate PA during recess *Secondary schools only* • Interscholastic sports offered	• Opportunities for PA during recess • Opportunities for unstructured PA time/free play • Incorporation of PA into the school day or during classroom-based instruction • Restrictions on use of PA as punishment • Policy restrictions on PA as a punishment • Opportunities for structured PA • Quality of structured PA • PA related elements of a school wellness policy
PA facilities (29)	• Students have access to loose play/exercise equipment, fixed play/exercise equipment, and/or stencils or game markings during recess or free time • PA, including PE, is held indoors during bad weather • Facilities are adequate to accommodate competing school/campus activities when they occur simultaneously • School PA facilities used by public when school is out of session • Active transport to school supported by specific safety features/supports on or near campus	• PA facilities, equipment, structures, or outdoor space • Playground markings/stencils to encourage PA • Access to exercise or recreation facilities • Access to PA facilities for after-hours recreation or shared use • Facility shared use agreement for PA • Access or safety of walking or bicycling paths • PA related elements of a school wellness policy
**Nutrition and PA practices** ^**3**^		
Wellness policies (8)	• School wellness policy reviewed, revised, or communicated to staff or families • Official responsible for school-level implementation and compliance with wellness policy • Meeting frequency of school-level wellness committee • Family engagement in school or district wellness committees • Wellness policy information shared with families	• Monitoring implementation of nutrition-related policies • Monitoring implementation of PA policies • Opportunities for parents or youth to participate in a wellness committee • Food/beverage or nutrition related elements of a school wellness policy • Policy limiting unhealthy foods • Policy increasing healthy foods and beverages • PA related elements of a school wellness policy

*SLAQ *Site-Level Assessment Questionnaire, *PEARS *Program Evaluation and Reporting System

^1^ For full questions and response options, see Supplementary Material 1, Additional File 1

^2^ Interventions refer to policy, systems, or environmental (PSE) changes being implemented, improved, expanded, or maintained; activities that support PSE change; or direct nutrition education classes implemented

^3^ Practices related to wellness policies were excluded from the nutrition and PA domains because they were not measured with enough specificity to assign them to either nutrition or PA

To determine if, and which type of interventions were implemented and therefore if a change in a given School SLAQ domain or practice area would be expected, nutrition and physical activity intervention data were extracted from the Program Evaluation and Reporting System (PEARS). PEARS is an online database used widely by SNAP-Ed implementers to report PSE and nutrition education interventions. PEARS data were extracted for federal fiscal year 2022 (10/1/2021-9/30/2022) in order to capture interventions that occurred between the two School SLAQ data collection timepoints. The research team monitored PEARS entries for inconsistent, missing, or implausible data and worked with local health department implementers to make corrections. Extracted PEARS data included stage of PSE implementation, PSE changes adopted, and nutrition education curriculum used. All education and PSE interventions were categorized as being related to nutrition, PA, or both, and further classified into nine SLAQ practice areas based on the policies and practices each intervention is likely to impact (see Table 1). In some cases, interventions were relevant to more than one practice area and were categorized as such. For example, the PSE intervention “policy increasing healthy foods and beverages” was categorized into both the meal environment and promotion and non-meal food and beverage quality and promotion practice areas. Changes in School SLAQ scores for a given domain or practice area were analyzed for the subset of schools that reported corresponding changes made in PEARS.

We used California Department of Education 2021-22 public school year data to determine schools’ grade levels, total student enrollment, and proportion of students who were eligible for free or reduced-price meals, identifying as Hispanic/Latino, and English language learners [20–22]. School urbanicity was obtained from the National Center for Education Statistics (2021-22) and categorized into urban, suburban, and town/rural [23]. 

### Data analysis

Descriptive analyses were conducted using California Department of Education and National Center for Education Statistics data. To assess the ability of the School SLAQ to detect change in school nutrition and PA practices in response to PSE interventions, Wilcoxon signed-rank and paired t-tests were conducted, assessing whether there were statistically significant changes in School SLAQ domain and practice area scores, respectively, from Year 1 to Year 2 among schools implementing relevant SNAP-Ed interventions. Standardized effect size (ES) estimates were then calculated to examine the magnitude of change. ES values were interpreted as follows: for Wilcoxon-signed rank test (r) - small (0.1 to < 0.3), medium (0.3 to < 0.5), and large ( > = 0.5); for paired t-test (Cohen’s dz) - small (0.2 to < 0.5), medium (0.5 to < 0.8), and large ( > = 0.8) [24–26]. All statistical tests were conducted using coin and rstatix packages in R (version 4.4.1). Results were considered statistically significant at *p* < 0.05.

## Results

Of the 103 SNAP-Ed-eligible California schools that completed School SLAQs in Year 1 and Year 2, 34 did not report PSE interventions for Year 1, and were thus excluded, yielding a final analytic sample of 69 schools. Schools had, on average, 379.8 days (SD = 69.5) between their Year 1 and Year 2 School SLAQ records. Most were elementary schools (87%), with nearly 50% located in urban areas (Table 2). On average, schools had 413 enrolled students, with 74% of students eligible for free or reduced-price meals, 57.9% identifying as Hispanic, and 40% English learners.

**Table 2:** Characteristics of schools in study sample (*n* = 69 schools)

**School-level characteristics**	***n*** **(%)**
* School type* ^*1*^	
Elementary school	60 (87.0)
Secondary school	9 (13.0)
* School urbanicity* ^*2*^	
Urban	37 (53.6)
Suburban	16 (23.2)
Town/rural	16 (23.2)
**Student-level characteristics**	**Mean (SD)**
Number of students enrolled^3^	413.5 (291.5)
Percent of students eligible for Free or Reduced Price Meals^1^	73.9 (19.6)
Percent of students identifying as Hispanic/Latino^3^	57.9 (27.4)
Percent of students who are English language learners^4^	39.3 (22.0)

^1^California Department of Education (CDE), Free or Reduced-Price Meal, 2021–2022

^2^National Center for Education Statistics, Education Demographic and Geographic Estimates, 2021–2022

^3^CDE, Enrollment by School 2020–2022

^4^CDE, English Learners by Grade & Language, 2021–2022

According to extracted PEARS data, nearly all schools implemented nutrition-related PSEs (94%), while about half implemented PA-related PSEs (52%), and about one in eight implemented wellness policy and/or wellness committee strategies (12%) (Table 3). Over two-thirds of schools (70%) implemented nutrition education classes. The top three PSE strategies adopted by schools were meal environment and promotion (83%), school meal and beverage quality (81%), and non-meal food and beverage quality and promotion (81%). Inversely, PSE strategies that were implemented by the fewest schools were physical education (20%), wellness policies (12%), and nutrition education professional development (1%). The most commonly used nutrition education curricula were the Nutrition Pathfinders Let’s Eat Healthy Video Series (25%) and the CATCH Kids Club Manual and Activity Box (19%).

**Table 3:** Intervention components^1^ implemented by schools in study sample in Federal Fiscal Year 2022 (*n* = 69 schools)

	**n (%)**
***Direct Nutrition Education***	**48 (69.6)**
* Nutrition-related curricula used*:	
Nutrition Pathfinders/Let’s Eat Healthy Video Series	17 (24.6)
CATCH - Kids Club Manual and Activity Box	13 (18.8)
TWIGS: Teams With Inter-Generational Support	9 (13.0)
Food Smarts for Kids	5 (7.2)
PowerPlay! Community Youth Organization Idea and Resource Kit	3 (4.3)
Let’s Eat Healthy First Grade	3 (4.3)
Growing Healthy Habits	3 (4.3)
Pick a Better Snack	3 (4.3)
Other curricula	16 (23.2)
**Policy**,** Systems**,** and Environmental (PSE) Change Strategies**	
**Wellness policy and committee PSE strategies adopted**	**8 (11.6)**
* Specific policy and committee strategies adopted*:	
Opportunities for parents or youth to participate in decision-making	8 (11.6)
Adoption, implementation, or monitoring of nutrition-related policy	4 (5.7)
Adoption, implementation, or monitoring of PA-related policy	4 (5.7)
**Any nutrition-related PSE strategies adopted**	**65 (94.2)**
* Nutrition-related PSE strategies adopted*:	
Meal environment & promotion	57 (82.6)
School meal & beverage quality	56 (81.2)
Non-meal food & beverage quality and promotion	56 (81.2)
Gardens	25 (36.2)
Nutrition education & professional development	1 (1.4)
**Any physical activity (PA)-related PSE strategies adopted**	**36 (52.2)**
* PA-related PSE strategies adopted*:	
PA facilities	29 (42.0)
Physical education (PE)	14 (20.3)
Non-PE PA opportunities	26 (37.7)

^1^Intervention data reported in the Program Evaluation and Reporting System (PEARS)

**Fig. 1:**
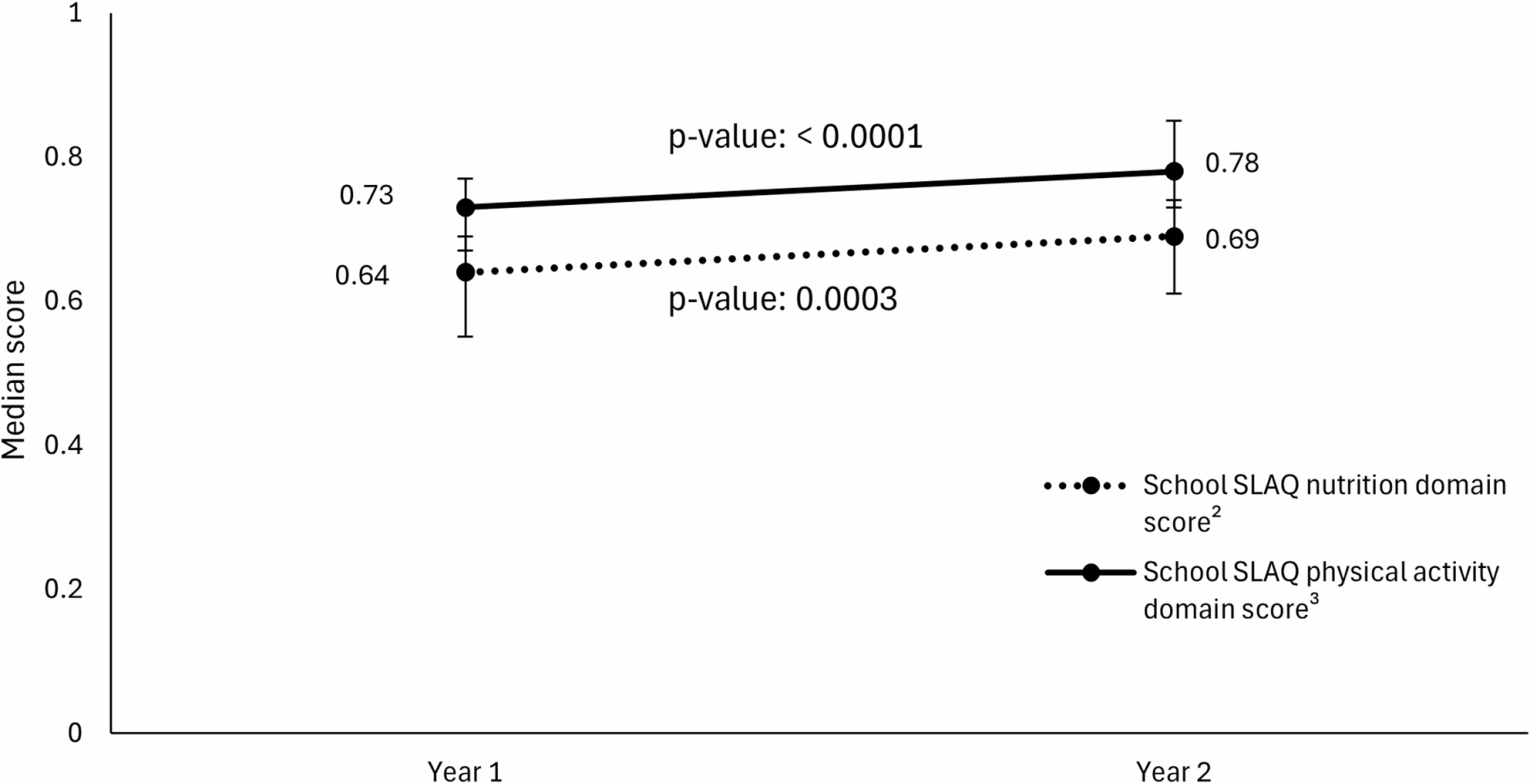
Change in median nutrition and PA School SLAQ domain scores between Years 1 and 2^1^. ^1^Among study sample schools implementing nutrition-related SNAP-Ed interventions (n=65 schools) or PA-related SNAP-Ed interventions (n=36 schools) in California in FFY22; PA: physical activity; Year 1 = school year 2021-22; Year 2 = school year 2022-23. ^2^Statistically significant change between Year 1 and Year 2 (p-value: 0.0003); Effect size = 0.42. ^3^Statistically significant change between Year 1 and Year 2 (p-value: <0.0001); Effect size = 0.64

There were statistically significant increases in both the School SLAQ nutrition and PA domain scores among schools that implemented relevant PSE interventions (Fig. 1). Specifically, a statistically significant increase in the median nutrition domain score of 0.06 (*p* < 0.001; ES = 0.42) from Year 1 to Year 2 was observed among schools that implemented nutrition-related PSEs in Year 1, and a statistically significant increase in the median PA domain score of 0.07 (*p* < 0.001; ES = 0.64) was observed among schools that implemented a PA-related PSE (Table 4).

**Table 4:** Changes in School SLAQ scores among California schools (*n* = 69) implementing corresponding SNAP-Ed interventions

		School SLAQ Scores (range 0–1)				
		Year 1	Year 2	Difference	*p*-value^3^	Effect size^4^
**Domain** ^1^	***n***	**Median (IQR)**				
Nutrition	65	0.64 (0.14)	0.69 (0.12)	0.06 (0.12)	**0.0003**	0.42
Physical activity	36	0.73 (0.10)	0.78 (0.12)	0.07 (0.08)	**< 0.0001**	0.64
**Practice area** ^**2**^	**n**	**Mean (SD)**		**Mean (95% CI)**		
Wellness policies	4	0.56 (0.11)	0.48 (0.19)	-0.08 (-0.25, 0.09)	0.215	-0.78
School meal & beverage quality	54	0.76 (0.17)	0.79 (0.14)	0.04 (0.002, 0.07)	**0.040**	0.29
Meal environment & promotion	47	0.67 (0.12)	0.72 (0.14)	0.04 (0.01, 0.08)	**0.020**	0.35
Non-meal food & beverage quality and promotion	45	0.70 (0.15)	0.79 (0.13)	0.09 (0.05, 0.13)	**< 0.001**	0.61
Garden	24	0.37 (0.34)	0.44 (0.31)	0.08 (-0.07, 0.22)	0.294	0.22
Nutrition education	43	0.55 (0.27)	0.65 (0.27)	0.10 (0.01, 0.18)	**0.031**	0.34
Physical education (PE)	13	0.67 (0.19)	0.70 (0.18)	0.04 (-0.06, 0.13)	0.443	0.22
Non-PE physical activity opportunities	23	0.66 (0.18)	0.76 (0.15)	0.09 (0.02, 0.17)	**0.034**	0.52
Physical activity facilities	28	0.68 (0.11)	0.75 (0.11)	0.07 (0.03, 0.12)	**0.003**	0.62

Year 1 = school year 2021-22; Year 2 = school year 2022-23

IQR Interquartile range, SD Standard deviation, 95% CI 95% Confidence Interval

^1^Wilcoxon signed-rank test was conducted to calculate Wilcoxon signed rank test statistic, z value, and corresponding p-value

^2^Paired t-test was conducted to calculate t-test statistic and corresponding p-value

^3^Statistically significant result at p-value < 0.05

^4^For Wilcoxon signed-rank test, 0.1 to < 0.3, 0.3 to < 0.5, and > = 0.5 were used as cut-offs for interpretation of small, moderate, and large effect size, respectively. For paired samples t-test, 0.2 to < 0.5, 0.5 to < 0.8, and > = 0.8 were used as cut-offs for interpretation of small, moderate, and large effect size, respectively

There were also statistically significant increases in six of the nine practice area scores (Table 4). Increases were detected from baseline to follow-up in three nutrition-related practice area mean scores among schools implementing corresponding SNAP-Ed PSE interventions: school meal & beverage quality (by 0.04 [95% CI: 0.002–0.07]), meal environment and promotion (by 0.04 [95% CI: 0.01–0.08]), and non-meal food and beverage quality and promotion (by 0.09 [95% CI:0.05–0.13]). Similarly, statistically significant increases were observed in two out of the three PA practice area mean scores among schools implementing corresponding SNAP-Ed PSE interventions: non-PE PA opportunities (by 0.09 [95% CI:0.02–0.17]) and PA facilities (by 0.07 [95% CI: 0.03–0.12]). Finally, a statistically significant increase in the nutrition education mean score of 0.10 (95% CI: 0.01–0.18) was observed among schools with nutrition education interventions, which could have included professional development in support of nutrition education and/or direct nutrition education. There were no statistically significant changes from Year 1 to Year 2 for garden, PE, and wellness policy practice areas. The effect size estimates for all the SLAQ practice area scores ranged from 0.22 (small) to 0.62 (moderate).

## Discussion

This study demonstrates that the School SLAQ was sensitive enough to detect change in nutrition and PA practices at K-12 schools that implemented corresponding interventions. In addition to detecting change in overall nutrition and overall PA practices, the instrument was also able to detect change in six of the nine specific practice areas measured: school meal and beverage quality, meal environment and promotion, non-meal food and beverage quality and promotion, nutrition education, non-PE PA opportunities, and PA facilities. The measured effect sizes ranged from small to moderate across these six practice areas, which while meaningful, may be even more pronounced when evaluated over a longer period than a single year. To our knowledge, this is the only validated instrument available to schools, public health practitioners, and researchers that has been tested for its ability to measure change over time in school nutrition and PA practices.

There were three practice areas for which statistically significant change was not detected at schools that reported corresponding interventions. This could indicate that the instrument is insufficiently sensitive to detect change in those areas, that those interventions were not adequate to create measurable change over a period of one year, that there was misalignment between interventions reported and the measured policies and practices, or that there was insufficient statistical power [27]. For two of these practice areas, gardens (*n* = 24) and PE (*n* = 13), a small positive effect was observed, but the small sample size of schools with corresponding interventions may have resulted in insufficient power to measure statistically significant change. The third practice area was wellness policy, for which only four schools in our study sample reported interventions with complete data. We were thus clearly underpowered to detect change within this practice area. Additionally, although both the assessment items and the corresponding intervention options were related to wellness policies, the assessment items covered specific facets of policy monitoring, implementation, and dissemination (e.g., wellness committee activities and membership), whereas the intervention options covered policy content more specifically (e.g., policy limiting unhealthy food) and monitoring and implementation more generally. Therefore, the SLAQ may not be able to detect the kinds of wellness policy-interventions reported in PEARS, i.e., specific policy content changes, even with a larger sample size. Intervention information that better corresponds to the specific policy monitoring and implementation efforts assessed by SLAQ would be needed to adequately test the sensitivity of this practice area.

Although this study demonstrated that the School SLAQ is sensitive to change for the domains of nutrition and PA, as well as for six of nine practice areas measured, it did not assess sensitivity at the item level for two reasons. First, sample sizes for individual items were too small for item-level analysis; for 51% of items, < 20 schools implemented the corresponding PSE intervention. Second, many (58%) of the School SLAQ items have categorical or trichotomous response options, considered too gross for item-level sensitivity [28]. While we constructed response options for these items knowing that item-level sensitivity would be limited, this was a necessary trade-off to measure these practices in a practical and logical manner; we therefore balanced these needs by grouping items to reflect practice areas, in order to achieve practice area-level sensitivity to change.

The School SLAQ is a self-report instrument, something that lends inherent strengths and limitations. While self-report can often result in biased reporting, prior research has shown that School SLAQ respondents willingly report sub-optimal practices, and as a result, we believe that self-report bias is likely to be small [11]. Moreover, in interpreting the results of this study, there is no reason to believe that self-report would systematically bias reporting of nutrition and physical activity practices in one study year more than the other. Schools, public health partners, and researchers considering use of the School SLAQ for planning and evaluation should consider the risks of self-report bias balanced with potential advantages. Notably, self-report enables the School SLAQ to efficiently capture the range of nutrition and physical activity practices far more comprehensively than more direct methods like observation or document review, and the process of engaging school administration and staff in assessment is known to be critical for translating assessment findings into effective and sustained programs [8]. 

This study had a couple of additional limitations because data were collected as part of annual SNAP-Ed reporting requirements. First, PEARS is only used to collect information about nutrition and physical activity interventions that are supported by SNAP-Ed funds. At some schools, there may be nutrition or physical activity programming on campus supported by other funding, and impacts of these programs may be reflected in practices reported on the School SLAQ. Similarly, schools are only asked to complete a School SLAQ when implementing PSE intervention. Consequently, this study does not have a comparison group to examine the specificity of the School SLAQ.

Despite these limitations, the results of this study suggest that the School SLAQ can be used for research and evaluation to sensitively assess changes at schools implementing nutrition and PA programming. The questionnaire detected relatively small changes in some practice areas, such as a 4% change between study years in practices related to school meal and beverage quality. Therefore, when using this instrument, researchers and practitioners should consider both statistical significance as well as practical significance of improvements to policies and programs. Schools using the School SLAQ to self-assess their nutrition and PA practices benefit from a validated tool that is designed for and successfully feasibility tested with school personnel and closely aligned with the Final Rule of the Healthy, Hunger-Free Kids Act of 2010 [10, 29]. In addition to using the School SLAQ to support planning and evaluation at individual school sites, local education agencies, public health organizations, and researchers can aggregate School SLAQ data from multiple sites to measure changes in nutrition and PA practices over time, such as to evaluate the effectiveness of organization- or community-level PSE interventions or to inform health policy.

For example, School SLAQ data collected from 153 elementary schools in California during the 2021-22 school year were analyzed by researchers at University of California, finding that only 56% of sampled schools reported implementing recess best practices, and that larger and lower-income schools were less likely to do so [30]. These data were shared with legislators and supported the passage of a state recess law that went into effect during the 2024-25 school year [31]. A follow-up study utilizing subsequent years of School SLAQ data could be used to evaluate the effectiveness of this state law in improving non-PE physical activity opportunities at schools and to support similar legislation in other states.

## Conclusions

The School SLAQ is a valid and reliable tool that comprehensively captures nutrition and physical activity related policies and practices in the school setting. It has been utilized by SNAP-Ed implementers to prioritize interventions that are likely to have the greatest impact on student health. This study demonstrates that the School SLAQ is able to detect changes in the school environment over time, which could be the result of school-based health promotion interventions or broader policy initiatives. Thus, the School SLAQ has utility not just as an assessment and planning tool, but also as an evaluation and research tool. Regular, comprehensive assessment of school practices, policies, and environments can be used to evaluate the impact of school-based programs, policies, and regulations as they emerge over time and further our understanding of which strategies are effective. Findings can be used to prioritize school-based interventions, particularly when resources are limited, and to generate an evidence base for policy changes that are likely to have the greatest impact on child health.

## Supplementary Information

Supplementary Material 1.

## Data Availability

The SLAQ data analyzed in this study are available from the corresponding author upon reasonable request. The PEARS data analyzed in this study were obtained and accessed through a contract with the California Department of Public Health. This dataset is available from the corresponding author upon reasonable request and with permission of the California Department of Public Health. The school urbanicity data used in this study are publicly available from the National Center for Education Statistics at [https://nces.ed.gov/programs/edge/Geographic/LocaleBoundaries](https:/nces.ed.gov/programs/edge/Geographic/LocaleBoundaries) . The school characteristics data used in this study are publicly available from the California Department of Education at [https://www.cde.ca.gov/ds/si/ds/pubschls.asp](https:/www.cde.ca.gov/ds/si/ds/pubschls.asp) .

## Abbreviations

PSE: Policy, systems, and environment(al)
PA: Physical activity
SLAQ: Site-Level Assessment Questionnaire
SNAP-Ed: Supplemental Nutrition Assistance Program-Education
PE: Physical education
PEARS: Program Evaluation and Reporting System
ES: Effect size
CI: Confidence interval
IQR: Interquartile range
