# Supplementary material for: Measuring change in school-based practices that promote children’s healthy eating and active living: a psychometric study
Source: Int J Behav Nutr Phys Act. 2026 Apr 17;23:53. doi: 10.1186/s12966-026-01921-0 (PMC13224441; doi:10.1186/s12966-026-01921-0)
Supplement: Supplementary file 1 — Supplementary Material 1. [file 12966_2026_1921_MOESM1_ESM.pdf]

Supplementary Material 1

Note: This version of the questionnaire reflects what was tested for this study. Please contact the corresponding author or visit this website for the latest version, which includes minor updates (e.g., removing specific mentions of COVID-19).

School Site-Level Assessment Questionnaire

Developed by the
University of California Nutrition Policy Institute for the
California Department of Public Health

Here is some basic information about the School Site-Level Assessment Questionnaire (SLAQ)

- **WHAT does it include?** Questions that assess current healthy eating and physical activity practices at a school site. Do NOT include practices that are planned but not yet implemented.
- **WHO should complete it?** One or more individuals that are familiar with the policies and practices in place at this school.
- **WHEN should this be completed?** Annually, *before* interventions begin for the school year at this site.
- **WHY?** To understand a school's need for healthy eating and physical activity supports, and to measure change and improvements over time.

Tips and additional information:

- Review the questionnaire before beginning to decide who should be involved in completing each and gather any materials/documents you may need.
- Do your best to estimate the current situation so that change over time can accurately be assessed. Do NOT include practices that are planned but not yet implemented.
- Current practices may be impacted by health or safety emergencies. It is important that you report practices in place at the time you complete the questionnaire, even though they may differ from the usual practices. There is a question at the end of each section to comment on these impacts. These questions are not included when computing scores.

This material was funded by USDA's Supplemental Nutrition Assistance Program – SNAP.

School Site Information

School name ____

School address ____

School district name ____

Date of current assessment (MM/DD/YYYY) ____________________

Which grade levels are <u>present at this school</u>? *Mark all that apply.*

☐ TK	☐ 4th	☐ 9th
☐ K	☐ 5th	☐ 10th
☐ 1st	☐ 6th	☐ 11th
☐ 2nd	☐ 7th	☐ 12th
☐ 3rd	☐ 8th	

This SLAQ <u>assesses the food and physical activity environment</u> of which grades:
Mark all that apply.

☐ TK	☐ 4th	☐ 9th
☐ K	☐ 5th	☐ 10th
☐ 1st	☐ 6th	☐ 11th
☐ 2nd	☐ 7th	☐ 12th
☐ 3rd	☐ 8th	

Section 1: Wellness Policies and Meal Program Participation

When completing this section, refer to policies about student wellness, NOT employee wellness. Unless otherwise specified, refer to practices in place currently. Do NOT include practices that are planned and not yet implemented.

1.1 During the past year, has anyone at your school done any of the following activities?
Mark all that apply.

☐ Reviewed your district's or school's wellness policy
☐ Helped revise your district's or school's wellness policy
☐ Communicated to school staff about your district's or school's wellness policy
☐ Communicated to parents and families about your district's or school's wellness policy
☐ None of these

1.2 Is there an official who is responsible for implementation and compliance with wellness policy at the school (not district) level?

○ Yes, a school employee
○ Yes, a district employee
○ No

1.3 Is there an active <u>school-level</u> wellness committee (an action-oriented advisory group of two or more people that focuses on the health and well-being of students)? IF SO, how often did it meet during the last 12 months?

○ Committee did not meet
○ 1-2 times
○ 3-4 times
○ 5-6 times
○ 7+ times

(Committee did not meet through 7+ times: *go to Q1.4*)

○ No school-level committee *skip to Q1.5*

1.4 Families from this school are active members of a school or district level wellness committee.

Mark all that apply.

☐ District level committee
☐ School level committee
☐ Neither
☐ Don't know

1.5 School participates in the National School Lunch Program. ○ Yes ○ No

1.6 School participates in the School Breakfast Program. ○ Yes ○ No

1.7 School participates in the Fresh Fruit & Vegetable Program.

○ Yes ○ No

○ N/A (middle/high school)

The Fresh Fruit and Vegetable Program (FFVP), administered by CDE, provides schools with funding to offer students a free fresh fruit or vegetable snack during the school day.

1.8 Have any of the policies or practices in Section 1 been impacted by COVID-19 or another emergency?

Mark all that apply.

☐ No

☐ Yes, impacted by COVID-19. *Please describe:*

____

☐ Yes, impacted by a wildfire. *Please describe:*

____

☐ Yes, impacted by another emergency. *Please describe:*

____

1.9 Comments on Section 1: Wellness Policies and Meal Program Participation

Add any notes or observations, such as a description of something asked in a question or additional practices that are not measured on this questionnaire.

____

____

____

1.10 Title(s) or role(s) of the people who completed Section 1:

____

Section 2: Meals and School Meal Environment

When completing this section, please refer to a weekly or monthly school meal menu.
Unless otherwise specified, refer to practices in place currently. Do NOT include practices that are planned and not yet implemented.

2.1 Which breakfast programs are offered at this school when students attend in-person?
Mark all that apply.

- ☐ N/A: no students attending in-person because of COVID-19
- ☐ Breakfast in the classroom
- ☐ "Grab and go" breakfast options (packaged, carry-away, reimbursable meal)
- ☐ "Second chance" breakfast (breakfast after homeroom or first period)
- ☐ This school offers breakfast but none of the above specific programs
- ☐ This school does not offer a breakfast program

2.2 The school participates in one or more programs that promote locally or regionally sourced food for meals.

- ○ Yes. *Please list:* ____
- ○ No

2.3 All foods and beverages provided in school meals meet nutritional requirements of the National School Lunch Act.

- ○ Always (true more than 90% of the time)
- ○ Usually (true 61-90% of the time)
- ○ Sometimes (true 41-60% of the time)
- ○ Not usually (true 11-40% of the time)
- ○ Never (true 10% or less of the time)

2.4 Students receive at least one fruit or vegetable with each meal.

- ○ Always (true more than 90% of the time)
- ○ Usually (true 61-90% of the time)
- ○ Sometimes (true 41-60% of the time)
- ○ Not usually (true 11-40% of the time)
- ○ Never (true 10% or less of the time)

2.5 Sliced or cut fruit is offered with meals.

- ○ Always (true more than 90% of the time)
- ○ Usually (true 61-90% of the time)
- ○ Sometimes (true 41-60% of the time)
- ○ Not usually (true 11-40% of the time)
- ○ Never (true 10% or less of the time)

2.6 Fruit and vegetables are prominently displayed in attractive containers on all service lines.

- ○ N/A: all meals are pre-packaged because of COVID-19
- ○ Always (true more than 90% of the time)
- ○ Usually (true 61-90% of the time)
- ○ Sometimes (true 41-60% of the time)
- ○ Not usually (true 11-40% of the time)
- ○ Never (true 10% or less of the time)

2.7 More than one fruit choice (not including juice) is offered *at each lunch*.

- ○ N/A: all meals are pre-packaged because of COVID-19
- ○ Always (true more than 90% of the time)
- ○ Usually (true 61-90% of the time)
- ○ Sometimes (true 41-60% of the time)
- ○ Not usually (true 11-40% of the time)
- ○ Never (true 10% or less of the time)

2.8 More than one vegetable choice is offered *at each lunch*.

- ○ N/A: all meals are pre-packaged because of COVID-19
- ○ Always (true more than 90% of the time)
- ○ Usually (true 61-90% of the time)
- ○ Sometimes (true 41-60% of the time)
- ○ Not usually (true 11-40% of the time)
- ○ Never (true 10% or less of the time)

2.9 Pre-packaged salads or a salad bar are available to all students *at lunch*.

- ○ Always (true more than 90% of the time)
- ○ Usually (true 61-90% of the time)
- ○ Sometimes (true 41-60% of the time)
- ○ Not usually (true 11-40% of the time)
- ○ Never (true 10% or less of the time)

2.10 The only beverages available to students at *breakfast* are milk and water.

- ○ N/A: no breakfast
- ○ Always (true more than 90% of the time)
- ○ Usually (true 61-90% of the time)
- ○ Sometimes (true 41-60% of the time)
- ○ Not usually (true 11-40% of the time)
- ○ Never (true 10% or less of the time)

2.11 The only beverages available to students at *lunch* are milk and water.

- ○ Always (true more than 90% of the time)
- ○ Usually (true 61-90% of the time)
- ○ Sometimes (true 41-60% of the time)
- ○ Not usually (true 11-40% of the time)
- ○ Never (true 10% or less of the time)

2.12 Milk served with meals is limited to non-flavored and 1% fat or less.

- ○ Always (true more than 90% of the time)
- ○ Usually (true 61-90% of the time)
- ○ Sometimes (true 41-60% of the time)
- ○ Not usually (true 11-40% of the time)
- ○ Never (true 10% or less of the time)

2.13 White milk is placed for easier access than flavored milk.

- ○ N/A: all meals are pre-packaged because of COVID-19
- ○ N/A: no flavored milk offered
- ○ Always (true more than 90% of the time)
- ○ Usually (true 61-90% of the time)
- ○ Sometimes (true 41-60% of the time)
- ○ Not usually (true 11-40% of the time)
- ○ Never (true 10% or less of the time)

2.14 This school provides access to free, palatable drinking water during meal times in the food service areas.

- ○ N/A: no on campus meal service because of COVID-19
- ○ Yes
- ○ No

2.15 Students have at least 30 minutes for eating lunch, including 20 minutes of "seat time" after getting their food. *Do not include time spent for recess during the lunch period.*

- ○ N/A: no on campus meal service because of COVID-19
- ○ Always (true more than 90% of the time)
- ○ Usually (true 61-90% of the time)
- ○ Sometimes (true 41-60% of the time)
- ○ Not usually (true 11-40% of the time)
- ○ Never (true 10% or less of the time)

2.16 Lunch (on campus) is served no earlier than 11:00 am and no later than 1:00 pm for:

- ○ N/A: no on campus meal service because of COVID-19
- ○ All students (true for more than 90% of students)
- ○ Most students (true for 61-90% of students)
- ○ Some students (true for 41-60% of students)
- ○ Few students (true for 11-40% of students)
- ○ No students (true for 10% or fewer students)

2.17 Classroom nutrition education is reinforced in the cafeteria with promotion such as posters, displays, taste testing, etc.

- ○ N/A: no on campus meal service because of COVID-19
- ○ N/A: not familiar with classroom nutrition education
- ○ More than once per month
- ○ About once per month
- ○ A few times per year
- ○ 1 time per year
- ○ Never

2.18 Students help promote the meal program. For example, students might make menu announcements or help develop materials promoting meals.

- ○ More than once per month
- ○ About once per month
- ○ A few times per year
- ○ 1 time per year
- ○ Never

2.19 Students take part in taste tests, surveys, or other methods to determine preferences for menu items.

- ○ More than once per month
- ○ About once per month
- ○ A few times per year
- ○ 1 time per year
- ○ Never

2.20 Students have adequate space to sit down and eat at a table most of the time.

- ○ N/A: no on campus meal service because of COVID-19
- ○ Yes, plenty of tables and extra space
- ○ Yes, but tables are crowded
- ○ No, not enough seating with tables

2.21 Dining facilities can be described as:

- ○ N/A: no on campus meal service because of COVID-19
- ○ Pleasant (very clean, well-lit, not too chaotic or noisy, and inviting)
- ○ Acceptable (adequately clean, well-kept, but sparse)
- ○ Some areas of concern (dirty, dingy, needs repairs, etc.)

2.22 Have any of the policies or practices in Section 2 been impacted by COVID-19 or another emergency?

Mark all that apply.

☐ No

☐ Yes, impacted by COVID-19. *Please describe:*

____

☐ Yes, impacted by a wildfire. *Please describe:*

____

☐ Yes, impacted by another emergency. *Please describe:*

____

2.23 Comments on Section 2: Meals and School Meal Environment

Add any notes or observations, such as a description of something asked in a question or additional practices that are not measured on this questionnaire.

____

____

____

2.24 Title(s) or role(s) of the people who completed Section 2:

____

Section 3: Food and Drink around the School

Unless otherwise specified, refer to practices in place currently. Do NOT include practices that are planned and not yet implemented.

3.1 Food and beverage advertising is limited to foods that meet the state and federal competitive food requirements.

- ○ N/A: students are not on campus because of COVID-19
- ○ N/A: no food and beverage advertising on campus
- ○ Yes
- ○ No
- ○ Don't Know

3.2 Items sold as part of fundraisers include:

- ○ Only items that meet the state and federal competitive food criteria (or include non-food items)
- ○ Some items that meet the state and federal competitive food criteria and some that do not
- ○ Only items that DO NOT meet the state and federal competitive food criteria

3.3 All foods and beverages *served* at school events and celebrations also meet state and federal competitive food standards.

- ○ N/A: no school events or celebrations because of COVID-19
- ○ N/A: no foods or beverages served at school events and celebrations
- ○ Yes
- ○ No

3.4 During this school year, less nutritious foods and beverages are intentionally priced at a higher cost relative to more nutritious foods and beverages.

- ○ N/A: students are not on campus because of COVID-19
- ○ N/A: no foods or beverages sold during the school day
- ○ Yes
- ○ No
- ○ Don't Know

3.5 All foods and beverages sold *during the school day* meet state and federal competitive food requirements. *Do not include reimbursable school meals or fundraisers.*

- ○ N/A: students are not on campus during the school day because of COVID-19
- ○ N/A: no foods or beverages sold during the school day
- ○ Yes
- ○ No

3.6 All foods and beverages sold on campus at *any time after school* meet state and federal competitive food standards. *Include sales that are on-going; do not include events/fundraisers that are once a year.*

- ○ N/A: students are not on campus after school because of COVID-19
- ○ N/A: no foods or beverages sold after school
- ○ Yes
- ○ No

3.7 *During the school day,* how many separate venues sell foods and beverages other than school meals? *Count each vending machine and all other points of sale on campus.*

- ○ N/A: students are not on campus during the school day because of COVID-19
- ○ N/A, this school serves elementary school students only
- ○ None
- ○ 1
- ○ 2
- ○ 3
- ○ 4 or more

3.8 Beverages sold to students on campus, not as part of meals, during the school day include:
Mark all that apply.

- ☐ N/A: students are not on campus during the school day because of COVID-19
- ☐ N/A: no foods or beverages sold during the school day
- ☐ Plain, unflavored water (carbonated or uncarbonated)
- ☐ Flavored water, no added sweeteners or caffeine (carbonated or uncarbonated)
- ☐ Unflavored low-fat (1%) or non-fat milk
- ☐ Unflavored whole or reduced fat (2%) milk
- ☐ Flavored non-fat milk
- ☐ Flavored whole, low-fat, or reduced fat milk
- ☐ 100% fruit or vegetable juices (full strength or diluted with water to include at least 50% juice)
- ☐ Calorie-free beverages with caffeine (e.g. unsweetened coffee, tea, or flavored waters with caffeine; excluding diet beverages and plain water)
- ☐ Low or no calorie electrolyte replacement beverages (< 5 calories per ounce such as low-calorie sports drinks)
- ☐ Regular, non-diet soda
- ☐ Diet drinks (including diet soda, light tea, artificially sweetened juice)
- ☐ Other sweetened drinks (including Capri Sun, fruit punch, lemonade, aguas frescas, sweet tea)
- ☐ Other (specify):

3.9 Are sugar-sweetened beverages served at school events?

Sugar-sweetened beverages include any drinks with added sugar, including corn syrup, like non-diet sodas, energy drinks, sports drinks, fruit drinks, and sweetened coffee or tea.

- ○ N/A: no school events because of COVID-19
- ○ Yes
- ○ No

3.10 Are teachers discouraged from serving sugar-sweetened beverages at classroom celebrations?

Sugar-sweetened beverages include any drinks with added sugar, including corn syrup, like non-diet sodas, energy drinks, sports drinks, fruit drinks, and sweetened coffee or tea.

- ○ N/A: students are not on campus because of COVID-19
- ○ N/A: no classroom celebrations because of COVID-19
- ○ Yes
- ○ No

3.11 Drinking water is available at no charge to students.

- ○ N/A: students are not on campus because of COVID-19
- ○ At all times and locations
- ○ At only some times or locations
- ○ No student access to drinking water

3.12 Students are allowed to carry refillable water bottles.

- ○ N/A: students are not on campus because of COVID-19
- ○ At all times and locations
- ○ At only some times or locations
- ○ Never

3.13 Students have access to water bottle fillers.

Do not count traditional drinking fountains, unless they have a water bottle filler feature.

- ○ N/A: students are not on campus because of COVID-19
- ○ No
- ○ Yes, students can access one water bottle filler
- ○ Yes, students can access two or more water bottle fillers

3.14 Have any of the policies or practices in Section 3 been impacted by COVID-19 or another emergency?

Mark all that apply.

☐ No

☐ Yes, impacted by COVID-19. *Please describe:*

____

☐ Yes, impacted by a wildfire. *Please describe:*

____

☐ Yes, impacted by another emergency. *Please describe:*

____

3.15 Comments on Section 3: Food and Drink around the School

Add any notes or observations, such as a description of something asked in a question or additional practices that are not measured on this questionnaire.

____

____

____

3.16 Title(s) or role(s) of the people who completed Section 3:

____

Section 4: Gardens

Unless otherwise specified, refer to practices in place currently. Do NOT include practices that are planned and not yet implemented.

4.1 During the last school year, did your school have access to an onsite or (offsite) community garden, used and maintained for growing fruits and vegetables? *Select the best option.*

- ○ Yes, we had access to an edible garden or planter that was in use and maintained for growing fruits or vegetables *Go to Q4.2*
- ○ No, the garden (or planter) was not being used to grow fruits or vegetables last year
- ○ No garden access

Skip to Q4.8

4.2 During the last school year, in which months was the garden actively growing fruits and/or vegetables? *Mark all that apply.*

☐ July	☐ August	☐ September	☐ October
☐ November	☐ December	☐ January	☐ February
☐ March	☐ April	☐ May	☐ June

When answering 4.3 to 4.7, think about the months the garden was actively growing fruits and/or vegetables in the last school year.

4.3 When nutrition education is offered the garden is incorporated:

Nutrition education refers to formal curriculum-based lessons.

- ○ Always (true more than 90% of the time)
- ○ Usually (true 61-90% of the time)
- ○ Sometimes (true 41-60% of the time)
- ○ Not usually (true 11-40% of the time)
- ○ Never (true 10% or less of the time)

4.4 Students tend to the garden:

"Tend to the garden" includes preparation for planting, weeding, watering, harvesting, etc.

- ○ All students (true for more than 90% of students)
- ○ Most students (true for 61-90% of students)
- ○ Some students (true for 41-60% of students)
- ○ Few students (true for 11-40% of students)
- ○ No students (true for 10% or fewer students)

4.5 Students tend to the garden:

"Tend to the garden" includes preparation for planting, weeding, watering, harvesting, etc.

- ○ 1 time per week or more
- ○ 2-3 times per month
- ○ 1 time per month
- ○ Less than 1 time per month
- ○ Never

4.6 Produce from the garden is distributed to families:

- ○ 1 time per week or more
- ○ 2-3 times per month
- ○ 1 time per month
- ○ Less than 1 time per month
- ○ Never

4.7 Produce from the garden is used in meals or snacks:

- ○ 1 time per week or more
- ○ 2-3 times per month
- ○ 1 time per month
- ○ Less than 1 time per month
- ○ Never

4.8 Have any of the policies or practices in Section 4 been impacted by COVID-19 or another emergency?

Mark all that apply.

- ☐ No
- ☐ Yes, impacted by COVID-19. *Please describe:*

  ____
- ☐ Yes, impacted by a wildfire. *Please describe:*

  ____
- ☐ Yes, impacted by another emergency. *Please describe:*

  ____

4.9 Comments on Section 4: Gardens

Add any notes or observations, such as a description of something asked in a question or additional practices that are not measured on this questionnaire.

____

4.10 Title(s) or role(s) of the people who completed Section 4:

____

Section 5: Nutrition Education

Nutrition education refers to formal curriculum-based lessons about nutrition, including drinking water. Unless otherwise specified, refer to practices in place currently. Do NOT include practices that are planned and not yet implemented.

5.1 This school offers nutrition education to students:

○ Yes, some or all students *Go to Q5.2*

○ No, no students *Skip to Q5.5*

5.2 How many students at each grade level receive nutrition education?

Select one response for each grade level present at the school.

	**Few/None** *<1/3 of students*	**Some** *1/3 to 2/3 of students*	**Most/All** *>2/3 of students*	**N/A** *Grade level not present*
TK	○	○	○	○
K	○	○	○	○
1st	○	○	○	○
2nd	○	○	○	○
3rd	○	○	○	○
4th	○	○	○	○
5th	○	○	○	○
6th	○	○	○	○
7th	○	○	○	○
8th	○	○	○	○
9th	○	○	○	○
10th	○	○	○	○
11th	○	○	○	○
12th	○	○	○	○

5.3 Nutrition education follows these practices: *Mark all that apply.*

☐ uses a standards-based curriculum with specific wellness goals

☐ is skills-based and participatory (i.e. practicing skills/behaviors such as goal setting, nutrition label reading, food preparation, menu planning, or media awareness)

☐ is integrated into other parts of the curriculum, e.g. in a math or science lesson

☐ is integrated with the broader school environment, e.g. a school cafeteria tour or offering school meal foods for taste tests in nutrition lessons

☐ incorporates agriculture and the food system, e.g. through gardening or a farm tour

☐ none of these

5.4 Teachers of nutrition education receive annual training/professional development in nutrition.

- ○ Yes
- ○ No

5.5 Have any of the policies or practices in Section 5 been impacted by COVID-19 or another emergency?

Mark all that apply.

- ☐ No
- ☐ Yes, impacted by COVID-19. *Please describe:*

  ____
- ☐ Yes, impacted by a wildfire. *Please describe:*

  ____
- ☐ Yes, impacted by another emergency. *Please describe:*

  ____

5.6 Comments on Section 5: Nutrition Education

Add any notes or observations, such as a description of something asked in a question or additional practices that are not measured on this questionnaire.

____

____

____

5.7 Title(s) or role(s) of the people who completed Section 5:

____

Section 6: Physical Education

Unless otherwise specified, refer to practices in place currently. Do NOT include practices that are planned and not yet implemented.

*Answer 6.1a for all **elementary school students** (those attending elementary schools OR schools serving grades 1-8).*

6.1a In a regular school week, how many minutes of Physical Education (PE) do elementary school students participate in, among those required to complete PE?

- ○ 150 minutes or more
- ○ 100-150 minutes
- ○ 99 minutes or less

*Answer 6.1b for all **middle and high school students** (those attending schools whose lowest grade is 6th or higher).*

6.1b Over 10 school days (two school weeks), how many minutes of Physical Education (PE) do secondary school students participate in, among those required to complete PE?

- ○ 450 minutes or more
- ○ 400-449 minutes
- ○ 399 minutes or less

6.2 At least 50% of PE class time is spent in moderate to vigorous physical activity.

- ○ Always (true more than 90% of the time)
- ○ Usually (true 61-90% of the time)
- ○ Sometimes (true 41-60% of the time)
- ○ Not usually (true 11-40% of the time)
- ○ Never (true 10% or less of the time)

6.3 PE curriculum as implemented aligns with California state PE standards, with grade-level benchmarks. *California state PE standards can be found here: https://www.cde.ca.gov/pd/ca/pe/physeducfaqs.asp*

- ○ Yes, for all grades
- ○ Yes, for some grades
- ○ No

6.4 The student-teacher ratio in PE classes is comparable to that in core classes.

- ○ Yes, for all classes
- ○ Yes, for some classes
- ○ No

6.5 PE is taught by a certified/endorsed PE teacher.

- ○ Yes, for all classes
- ○ Yes, for some classes
- ○ No

6.6 Teachers of PE complete annual professional development on PE or physical activity.

- ○ Yes
- ○ No

6.7 Have any of the policies or practices in Section 6 been impacted by COVID-19 or another emergency?

Mark all that apply.

☐ No

☐ Yes, impacted by COVID-19. *Please describe:*

____

☐ Yes, impacted by a wildfire. *Please describe:*

____

☐ Yes, impacted by another emergency. *Please describe:*

____

6.8 Comments on Section 6: Physical Education

Add any notes or observations, such as a description of something asked in a question or additional practices that are not measured on this questionnaire.

____

____

____

6.9 Title(s) or role(s) of the people who completed Section 6:

____

Section 7: Physical Activity

Unless otherwise specified, refer to practices in place currently. Do NOT include practices that are planned and not yet implemented.

7.1 In addition to a lunch break, recess is provided for all students:

- ○ N/A: This school's lowest grade is 6th or higher *Skip to Q7.4*
- ○ More than 20 min per day
- ○ 20 min per day
- ○ 10-19 min per day
- ○ <10 min per day
- ○ Never *Skip to Q7.3*

Go to Q7.2 (More than 20 min per day; 20 min per day; 10-19 min per day; <10 min per day)

7.2 Staff actively facilitate physical activity during recess.

- ○ N/A: Staff and students are not on campus because of COVID-19
- ○ Always (true more than 90% of the time)
- ○ Usually (true 61-90% of the time)
- ○ Sometimes (true 41-60% of the time)
- ○ Not usually (true 11-40% of the time)
- ○ Never (true 10% or less of the time)

7.3 Free time during, before, and/or after school (including recess) is characterized by the provision of: *Mark all that apply.*

- ☐ N/A: Students are not on campus because of COVID-19
- ☐ ample loose equipment (e.g., balls, jump ropes, hula hoops)
- ☐ fixed sports and play equipment (e.g., climbing walls, slides, monkey bars, basketball hoops, soccer goals, volleyball nets, stationary bikes)
- ☐ strategic playground or game markings (e.g., stencils, four square, hopscotch)
- ☐ none of these

7.4 Teachers are encouraged to provide movement breaks throughout the day.

- ○ Yes
- ○ No

7.5 Teachers are deterred (discouraged or prevented) from using or withholding physical activity to punish or manage behavior.

- ○ Yes
- ○ No

7.6 The school offers physical activity, including PE, indoors when bad weather prohibits outdoor activities.

- ○ N/A: Students are not on campus because of COVID-19
- ○ Always (true more than 90% of the time)
- ○ Usually (true 61-90% of the time)
- ○ Sometimes (true 41-60% of the time)
- ○ Not usually (true 11-40% of the time)
- ○ Never (true 10% or less of the time)

7.7 Indoor and outdoor spaces (gym, multipurpose, locker rooms) are adequate to accommodate the needs of PE classes, recess, team practices, and other activities when they occur simultaneously.

- ○ N/A: These classes or activities are not on campus because of COVID-19
- ○ Always (true more than 90% of the time)
- ○ Usually (true 61-90% of the time)
- ○ Sometimes (true 41-60% of the time)
- ○ Not usually (true 11-40% of the time)
- ○ Never (true 10% or less of the time)

7.8 The school offers opportunities for students to participate in organized physical activities during the following times. *Mark all that apply.*

- ☐ N/A: Students are not on campus before or after school because of COVID-19
- ☐ Before school
- ☐ After school
- ☐ Neither

7.9 Intramural sports programs or physical activity clubs are offered for students regardless of gender and ability.

Intramural sports are programs organized within a school for recreation or competition.

- ○ N/A: there are no student activities because of COVID-19
- ○ Yes, for all grades
- ○ Yes, for some grades
- ○ No

7.10 The school offers interscholastic sports to students.

Interscholastic sports are school-sponsored sports with between-school competition.

- ○ Yes, for all grades
- ○ Yes, for some grades
- ○ No
- ○ N/A: elementary school

7.11 School grounds/physical activity facilities are used by the public when school is not in session:

- ○ More than once a week
- ○ About once a week
- ○ Never or rarely

7.12 Active transport to school is supported by the following safety features or other supports on or near the school campus. *Active transport means using non-motorized forms of transportation, such as walking and cycling. For longer distance trips, it may also include public transit, as these trips may include walking or cycling for part of the trip. These practices are sometimes supported by "Safe Routes to School."*

Mark all that apply.

- ☐ Sufficient, well-marked crosswalks around the school
- ☐ Sufficient crossing guards around the school
- ☐ Speed bumps/traffic calming on school grounds or neighboring streets
- ☐ Adequate bike racks on the school campus
- ☐ Organized walk-to-school groups, such as a "walking school bus"
- ☐ Other (specify):
- ☐ No active transport supports are currently in place

7.13 Have any of the policies or practices in Section 7 been impacted by COVID-19 or another emergency?

Mark all that apply.

- ☐ No
- ☐ Yes, impacted by COVID-19. *Please describe:*
  ____
- ☐ Yes, impacted by a wildfire. *Please describe:*
  ____
- ☐ Yes, impacted by another emergency. *Please describe:*
  ____

7.14 Comments on Section 7: Physical Activity

Add any notes or observations, such as a description of something asked in a question or additional practices that are not measured on this questionnaire.

____

____

____

7.15 Title(s) or role(s) of the people who completed Section 7:

____

Section 8: Parent and Family Involvement

Unless otherwise specified, refer to practices in place currently. Do NOT include practices that are planned and not yet implemented.

8.1 The school refers parents and families to community-based nutrition and physical activity services and programs by:

Mark all that apply.

- ☐ Active referrals (such as outreach from a family resource staff member)
- ☐ Materials provided (such as program information distributed to students or available on school website, brochures displayed in the school office)
- ☐ None of the above

8.2 Nutrition education (workshops, activities, and take-home materials) is offered to parents and caregivers:

- ○ More than 1 time per year
- ○ 1 time per year
- ○ Less than 1 time per year or never *Skip to Q8.3*

8.3 Physical activity opportunities or promotions (workshops, activities, and take-home materials) are offered to parents and caregivers:

- ○ More than 1 time per year
- ○ 1 time per year
- ○ Less than 1 time per year or never *Skip to Q8.4*

8.4 The nutritional content of foods and beverages served to students is made available to families (sent home or posted online):

- ○ Yes
- ○ No

8.5 Easy-to-understand information about wellness policies are distributed to all parents at least annually:

- ○ Yes
- ○ No

8.6 Guidelines for food brought in for holidays or celebrations are provided to families at least once a year:

- ○ Yes
- ○ No

8.7 Information is provided to families about how to enroll students in physical activity opportunities on campus before and after school.

- ○ N/A: students are not on campus before or after school because of COVID-19
- ○ Yes
- ○ No

8.8 Have any of the policies or practices in Section 8 been impacted by COVID-19 or another emergency?

Mark all that apply.

☐ No

☐ Yes, impacted by COVID-19. *Please describe:*

____

☐ Yes, impacted by a wildfire. *Please describe:*

____

☐ Yes, impacted by another emergency. *Please describe:*

____

8.9 Comments on Section 8: Parent and Family Involvement

Add any notes or observations, such as a description of something asked in a question or additional practices that are not measured on this questionnaire.

____

____

____

8.10 Title(s) or role(s) of the people who completed Section 8:

____

Thank you for completing the School SLAQ!
